# Intravitreal Aflibercept for the Treatment of Diabetic Retinopathy Among Patients Who Completed PANORAMA: 1-Year Outcomes from the VOYAGE Extension Study

**DOI:** 10.3390/jpm15110555

**Published:** 2025-11-14

**Authors:** Avery W. Zhou, Gail M. Teagle, Liisa M. Baumann, Jessica A. Cao, Andres Emanuelli, Allen Y. Hu, Adam S. Berger, James C. Major, Seong Y. Lee, Stephen M. Huddleston, Victor H. Gonzalez, W. Lloyd Clark, David S. Liao, Ronald M. Kingsley, Howard S. Lazarus, John F. Payne, Eric G. Feinstein, Annal D. Meleth, Sagar B. Patel, Kenneth C. Fan, Alyson J. Berliner, Hadi Moini, Xiaomeng Niu, Michael S. Ip, SriniVas R. Sadda, Hasenin Al-khersan, Charles C. Wykoff

**Affiliations:** 1 Retina Consultants of Texas, Retina Consultants of America, Bellaire, TX 77401, USAgail.teagle@retinaconsultantstexas.com (G.M.T.); liisa.baumann@retinaconsultantstexas.com (L.M.B.); jessica.cao@utsouthwestern.edu (J.A.C.);; 2 Emanuelli Research and Development Center, Arecibo 00612, Puerto Rico; 3 Cumberland Valley Retina Consultants, Hagerstown, MD 21740, USA; 4 Center for Retina and Macular Disease, Clermont, FL 34711, USA; 5 Strategic Clinical Research Group, Willow Park, TX 76087, USA; 6 Charles Retina Institute, Germantown, TN 38138, USA; stevehudd@gmail.com; 7 Valley Retina Institute, McAllen, TX 78503, USA; 8 North Carolina Retina Associates, Retina Consultants of America, Raleigh, NC 27609, USA; 9 Retina-Vitreous Associates Medical Group, Los Angeles, CA 90017, USA; 10 Dean McGee Eye Institute, Oklahoma City, OK 73104, USA; ronald-kingsley@dmei.org; 11 John-Kenyon American Eye Institute, Louisville, KY 40222, USA; hlazarus@johnkenyon.com; 12 Palmetto Retina Center, Retina Consultants of America, Columbia, SC 29204, USA; 13 Central Florida Retina, Orlando, FL 32806, USA; 14 Marietta Eye Clinic, Marietta, GA 30060, USA; 15 Regeneron Pharmaceuticals, Inc., Tarrytown, NY 10591, USA; 16 Doheny Eye Institute, Pasadena, CA 91103, USA

**Keywords:** aflibercept, diabetic retinopathy, anti-VEGF, extension study

## Abstract

**Background/Objectives**: Evaluate outcomes and treatment patterns with 2 mg intravitreal aflibercept injection among patients who completed the phase 3 PANORAMA trial and enrolled in the VOYAGE (ClinicalTrials.gov identifier, NCT04708145; 12 January 2021) long-term extension study. **Methods**: During VOYAGE, patients were evaluated every 16 weeks and treated with 2 mg intravitreal aflibercept injection as needed depending on ophthalmoscopic examination findings. Those with no history of panretinal photocoagulation (PRP) received aflibercept if their clinician-determined diabetic retinopathy severity scale (DRSS) level was ≥47, corresponding to moderately severe non-proliferative diabetic retinopathy (NPDR). Patients with a history of PRP received aflibercept if active neovascularization was present. New or worsening diabetic retinopathy (DR) severity prompted more frequent treatment. **Results**: 320 patients (1 eye per patient) from 87 sites completed the PANORAMA trial. Of these, 41 patients (13% of PANORAMA completers) from 14 sites (16%) enrolled in VOYAGE after a mean interim period of 33.7 months, and 35 patients (85%) completed study visits through 1 year. At year 1 in VOYAGE, the mean number of anti-vascular endothelial growth factor (VEGF) injections increased from 1.1 per year during the interim period to 3.4 per year and was associated with stabilization or improvement in DRSS level in 81% (26/32) of patients. Mean best-corrected visual acuity (BCVA) remained relatively stable, and mean central subfield thickness (CST) improved by 24.4 µm to 269.5 μm through year 1 of VOYAGE. There were no unexpected safety events. **Conclusions**: Following a mean of 3 years of routine clinical care with associated declines in DRSS level, CST, and BCVA, stabilization of DRSS level and BCVA with reductions in CST was achieved through year 1 of the VOYAGE extension study, with a concurrent increase in aflibercept dosing frequency.

## 1. Introduction

Diabetic retinopathy (DR) remains a leading cause of global vision loss and is estimated to affect 9.6 million people in the United States alone [1]. Vision-threatening complications from DR include proliferative DR (PDR) and diabetic macular edema (DME) [2]. Diabetic retinopathy severity can be assessed using structured grading of fundus color images based on the Early Treatment Diabetic Retinopathy Study (ETDRS) severity scale [3]. There are 12 steps within the Diabetic Retinopathy Severity Scale (DRSS): levels 10 and 20 represent no DR and very mild DR, respectively; levels 35 and 43 represent mild and moderate non-proliferative DR (NPDR), respectively; levels 47 and 53 represent moderately severe and severe NPDR, respectively; and levels 60, 61, 65, 71, 75, 81, and 85 represent increasing severities of PDR [3]. A higher level within the NPDR portion of the ETDRS DRSS is highly predictive of progression to PDR [4], a finding confirmed in multiple recent prospective clinical trials [5,6].

Anti-vascular endothelial growth factor (VEGF) pharmacotherapy has demonstrated the ability to slow DR progression, reduce DME and PDR development, and improve DRSS levels in a meaningful proportion of patients [6,7]. The randomized, double-masked, sham-controlled 100-week, phase 3 PANORAMA (NCT02718326) trial evaluated 2 mg intravitreal aflibercept injection in patients with moderately severe to severe NPDR (DRSS levels 47 and 53) [6]. In PANORAMA, 402 patients (1 eye per patient) with best-corrected visual acuity (BCVA) of 20/40 or better were randomized to either sham, aflibercept every 16 weeks following 3 initial monthly doses and one 8-week interval (aflibercept 2q16), or aflibercept every 8 weeks following 5 initial monthly doses with pro re nata (PRN) dosing in year 2 if DRSS level was worse than 35 (aflibercept 2q8/PRN). Findings from PANORAMA revealed a significantly greater proportion of patients in the aflibercept 2q8/PRN and 2q16 groups experienced a ≥ 2-step DRSS level improvement from baseline at week 52 compared with those in the sham group (80% and 65% versus 15%, respectively), and these improvements were maintained through week 100. Most clinically relevant, fewer patients treated with aflibercept versus sham experienced development of PDR or DME through week 100.

For many patients, DR and its associated manifestations is a chronic disease, and long-term close monitoring and personalized care are needed to achieve optimal clinical outcomes [8,9,10]. However, data on long-term outcomes are limited among patients with NPDR without DME receiving anti-VEGF injections. The VOYAGE phase 4 open-label PANORAMA extension study was therefore conducted to evaluate the long-term outcomes of patients who completed PANORAMA after an intervening period of routine clinical care and help contribute to our understanding of the long-term efficacy and safety of anti-VEGF treatment among these patients.

## 2. Materials and Methods

VOYAGE (ClinicalTrials.gov identifier, NCT04708145; 12 January 2021) was a prospective, phase 4 open-label extension study which included patients who completed the preceding phase 3 PANORAMA trial. The study protocol was approved by the Advarra Institutional Review Board and Ethics Committee at each participating site, and the study was performed in adherence with the principles of the Declaration of Helsinki and local regulations [11]. All patients provided written informed consent. Data from patients between the end of PANORAMA and VOYAGE enrollment (the interim period) were retrospectively collected to evaluate patterns of treatment and clinical outcomes during this phase. Collected data included demographic information, visit dates, original randomized groups in PANORAMA, anti-VEGF treatments, laser procedures such as panretinal photocoagulation (PRP), phakic status, BCVA, central subfield thickness (CST), DRSS level, and glycosylated hemoglobin (HbA1c) when available.

During VOYAGE, patients were treated with 2 mg intravitreal aflibercept injection PRN depending on ophthalmoscopic examination findings and investigator-determined DRSS level. Based on the presence or absence of prior PRP application at VOYAGE enrollment, patients were categorized into Group 1, eyes without PRP, or Group 2, eyes with PRP.

Patients with study eyes without PRP (Group 1) were evaluated every 16 weeks and treated with 2 mg intravitreal aflibercept injection if the clinician-determined DRSS level was 47 (moderately severe NPDR) or worse; patients with DRSS level < 47 were not treated. The interval between visits was decreased to every 8 weeks if DRSS level was ≥ 53, there was ≥2-step DRSS level worsening compared to the last protocol-scheduled 16-week visit, or if the patient developed active PDR. The patient could then be treated with aflibercept every 8 weeks until there was no active PDR and DRSS improved to the level observed at the visit before the patient began being seen at 8-week intervals.

Patients with study eyes with PRP (Group 2) were evaluated every 16 weeks and treated with 2 mg intravitreal aflibercept injection if the neovascular disease process was active and stable. If the neovascular process was determined to be inactive, no treatment was given. If new or worsening neovascular disease was present, patients were treated every 8 weeks until the process became stable or inactive, at which time the interval between visits increased to every 16 weeks.

If high-risk PDR (defined as neovascularization of the disc greater than 1/3 disc area or any neovascularization associated with vitreous or preretinal hemorrhage) developed, PRP therapy could be applied at the investigator’s discretion, although PRP was not required, and investigators were allowed to use aflibercept monotherapy for PDR management. If vitreous or preretinal hemorrhage developed, aflibercept could have been given as often as every 4 weeks until hemorrhage improved and was no longer considered vision-threatening. Patients who developed clinically relevant DME (defined as CST > 320 μm on optical coherence tomography [OCT] or clinically worse than baseline with associated vision loss) could have been treated with aflibercept every 4 or 8 weeks at the investigator’s discretion until the DME resolved.

At all visits, patients underwent ETDRS BCVA testing at 4 m, slit lamp and dilated ophthalmic examination, and spectral-domain OCT imaging. Ultra-widefield fundus photography was performed every 16 weeks and was used to determine DRSS levels and presence of center-involving DME by a central reading center (Doheny Image Reading Center, Los Angeles, CA, USA). Per the reading center protocol, eyes could be graded as DRSS level < 61 if there were no active PDR signs, regardless of the presence of PRP scars.

The objective of VOYAGE was to assess longitudinal DRSS level changes, and the primary efficacy endpoint was the proportion of patients achieving DRSS level of 43 (moderate NPDR) or better at 1 year. Secondary outcomes were the proportion of patients with stable, worsened, or improved DRSS level; mean and annualized aflibercept injection frequencies; mean change in ETDRS BCVA; mean change in CST; and incidence of ocular and systemic adverse events (AEs).

All analyses used observed data. The denominators for percentages varied depending on the total data available for that variable. Group comparisons were based on two-tailed *t*-tests, paired where applicable, or analysis of variance (ANOVA) for continuous variables, and chi-square tests for categorical variables. Statistical analyses were performed using Microsoft Excel 2016 (Microsoft Corp, Redmond, WA, USA) and GraphPad Prism version 10.6.0 for Windows (GraphPad Software, Boston, MA, USA, www.graphpad.com [accessed on 25 August 2025]).

## 3. Results

**Figure 1:**
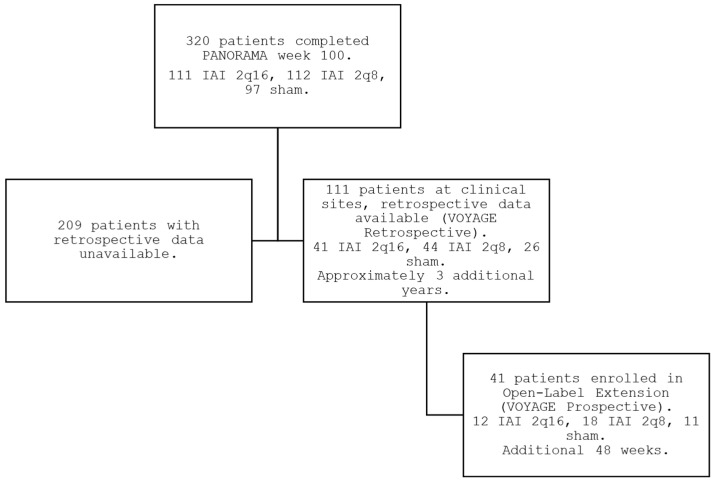
PANORAMA, interim period, and VOYAGE study flow.

Of the 87 sites that participated in the core PANORAMA phase 3 trial, 14 sites (16%) participated in VOYAGE and enrolled patients in the prospective VOYAGE extension study (full list of participating sites in Table S1). Of the 111 patients completing PANORAMA at these sites who were potentially eligible for enrollment, 41 patients (37%) enrolled into VOYAGE from June 2021 to July 2022, constituting 13% of the total 320 patients who completed PANORAMA (Figure 1). The demographics of the VOYAGE patients were consistent with those of the overall PANORAMA population, with all 3 original randomized arms being approximately equally represented within VOYAGE (Table 1). The total mean (SD) time of follow-up from initial enrollment in PANORAMA to the 1-year endpoint of VOYAGE was 5.7 years (0.4), or approximately 5 years and 8 months.

### 3.1. Clinical Course During Interim Period

**Figure 2:**
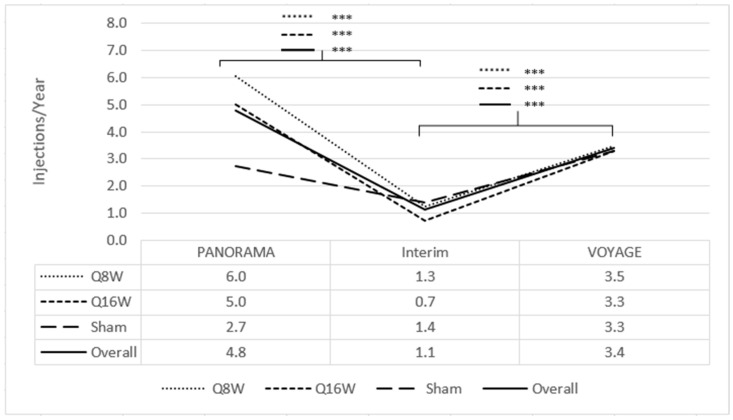
Mean injection frequency from PANORAMA enrollment to VOYAGE 1-year, annualized. The line corresponding to *** signifies statistical significance of <0.001 on a two-tailed *t*-test.

Among the 41 patients enrolled, the mean length of the interim period from PANORAMA exit to VOYAGE enrollment was 33.7 months (SD 4.8, range 23.9–44.2). During this period, 24 patients (59%, 24/41) received no anti-VEGF injections, while the other 17 patients (41%, 17/41) received a mean of 2.7 anti-VEGF injections per year. Among all patients, a mean of 1.1 injections per year (SD 1.9) were given during the interim period (Figure 2). Patients who had been in the combined aflibercept arms within PANORAMA (*n* = 30) experienced significant decreases in annualized mean anti-VEGF injections from 5.6 injections per year (SD 1.3) during PANORAMA to 1.0 injections per year (SD 1.6) in the interim (*p* < 0.001), whereas sham patients within PANORAMA (*n* = 11) did not have significantly different anti-VEGF injection rates during PANORAMA due to the institution of rescue therapy (2.7 injections per year [SD 3.9]) compared to the interim period (1.4 injections per year [SD 2.6]) (*p* = 0.36).

Three patients (7%, 3/41) received PRP during the interim period. Nine patients (22%, 9/41) received focal macular laser during the interim period. One patient (2%, 1/41) underwent pars plana vitrectomy (PPV) during the interim period for vitreous hemorrhage.

**Figure 3:**
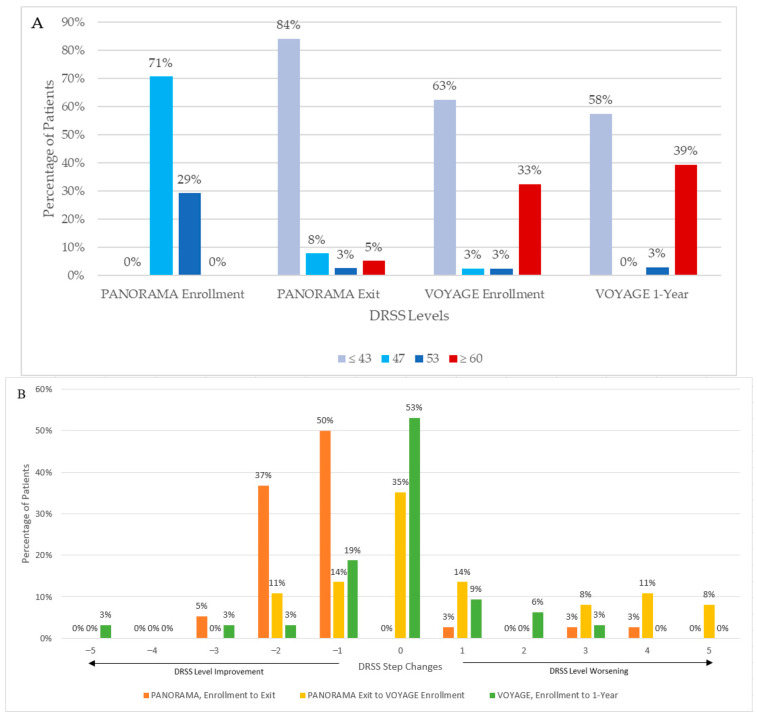
Diabetic Retinopathy Severity Scale (DRSS) levels from PANORAMA enrollment to VOYAGE 1-year. (**A**) Absolute DRSS levels. (**B**) Step changes in DRSS levels; positive values represent DRSS step worsening to a greater severity, and negative values represent DRSS step improvements to a lower severity.

At PANORAMA exit, 73% (8/11) and 89% (24/27) of the sham and combined aflibercept arms had DRSS level ≤ 43, respectively. During the interim period, 41% (15/37) of patients worsened, of which 5 patients worsened 1 step and 10 patients worsened ≥ 2 steps; 13 patients (35%, 13/37) had stable DRSS levels; and 9 (24%, 9/37) improved by 1 or 2 steps at VOYAGE enrollment compared to PANORAMA exit (Figure 3). Notably, the proportion of patients with PDR (DRSS level ≥ 60) increased from 5% (2/38) to 33% (13/40), while the proportion of patients with DRSS level ≤ 43 decreased from 84% (32/38) at the beginning of the interim period to 63% (25/40) at the end. Additionally, mean HbA1c remained stable during the interim period (8.9% [SD 1.8] at PANORAMA exit and 8.9% [SD 2.4] at VOYAGE enrollment). Seven patients (18%, 7/39) had center-involving DME by the end of the interim period.

Sub-analysis of the 22 patients (59%, 22/37) who experienced stable or improved DRSS levels during the interim period revealed a roughly equal distribution of patients assigned to PANORAMA Q16W, Q8W, and sham arms (5:10:7); 15 of the 22 patients (68%) had a PANORAMA baseline DRSS level of 47, and the other 7 patients (32%) had a baseline DRSS level of 53. Of the patients with stable or improved DRSS levels during the interim period, 20 patients (91%, 20/22) were in Group 1, and the other 2 patients (9%, 2/22) were in Group 2. This is a similar distribution as that of the 15 patients who experienced worsening DRSS levels during the interim period, with 87% (13/15) in Group 1 and 13% (2/15) in Group 2. There was no meaningful difference in the proportion of patients who improved, remained stable, or worsened during VOYAGE compared with their previous mean anti-VEGF injection frequency during PANORAMA (one-way ANOVA, *p* > 0.05). There were slight differences during the interim period (one-way ANOVA with multiple comparisons, *p* = 0.04): 2.1 greater annual injections for patients who improved compared to remained stable (*p* = 0.04), and 1.8 greater annual injections for patients who improved compared to worsened (*p* > 0.05), while patients who remained stable compared to worsened received approximately the same number of injections (*p* > 0.05).

**Figure 4:**
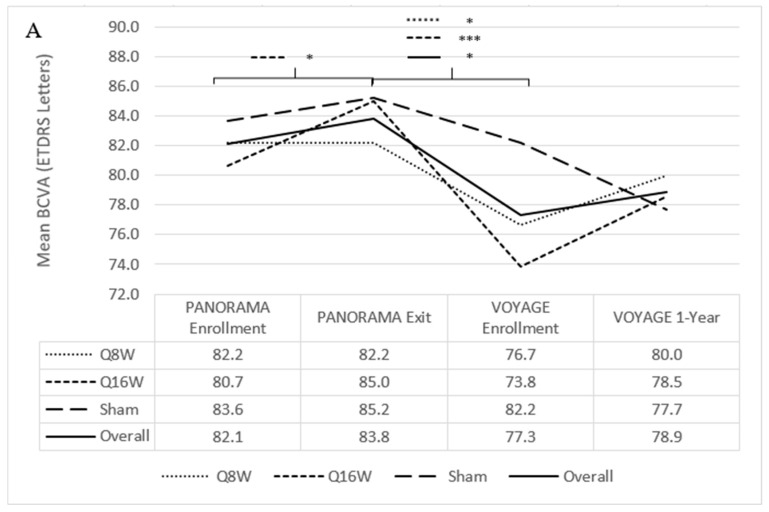

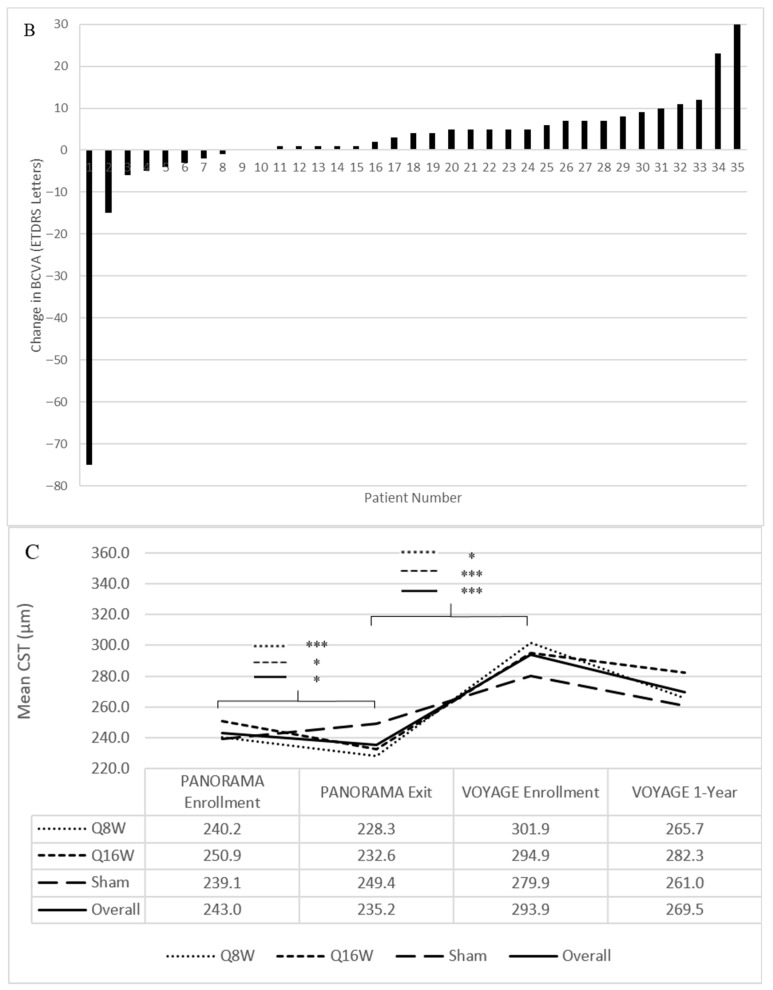
Mean best-corrected visual acuity (BCVA) and central subfield thickness (CST) from PANORAMA enrollment to VOYAGE 1-year. (**A**) Mean BCVA from PANORAMA enrollment to VOYAGE 1-year. (**B**) Change in BCVA from VOYAGE baseline to VOYAGE 1-year for each patient. Notably, patients 1, 2, 29, and 35 had a traumatic cataract resulting in 0 letters read at VOYAGE 1-year, endophthalmitis resulting in a loss of 15 letters by VOYAGE 1-year, cataract extraction resulting in an improvement of 8 letters at VOYAGE 1-year, and vitreous hemorrhage resulting in 0 letters read at VOYAGE baseline, respectively. (**C**) Mean CST from PANORAMA enrollment to VOYAGE 1-year. ETDRS = Early Treatment Diabetic Retinopathy Study. Line-corresponding * signifies statistical significance of *p* < 0.05, and *** signifies statistical significance of *p* < 0.001 on a two-tailed *t*-test.

At PANORAMA exit, mean BCVA was not significantly different between the combined aflibercept and sham arms. Mean BCVA decreased during the interim period from 83.8 letters (SD 6.1) to 77.3 letters (SD 14.8) (*p* = 0.009), representing a mean loss of 6.5 letters (Figure 4A). BCVA losses during the interim period were most notable among patients who had been in the combined aflibercept arms, with combined mean BCVA decreasing by 7.8 mean letters, from 83.3 letters (SD 5.9) at PANORAMA exit to 75.5 letters (SD 16.6) at VOYAGE enrollment (*p* = 0.02). BCVA losses were less among patients previously enrolled in the sham arm (85.2 letters [SD 6.6] to 82.2 letters [SD 6.8]). Patients from all of the PANORAMA arms combined who remained in the NPDR stage (*n* = 26, DRSS level ≤ 53) throughout the interim period also experienced a mean 4.3 letter loss from 85.5 letters (SD 4.2) at PANORAMA exit to 81.2 letters (7.0) at VOYAGE enrollment (paired, *p* < 0.001).

At PANORAMA exit, mean CST was not significantly different between the combined aflibercept and sham arms. Mean CST during the interim period increased from 235.2 μm (SD 39.1) to 293.9 μm (SD 67.9) (*p* < 0.001) (Figure 4C). Changes in CST during the interim period were most notable among patients who had been in the combined aflibercept arms, with combined mean CST increasing by 69.2 μm, from 230.0 μm (SD 32.6) to 299.2 μm (SD 73.5) (*p* < 0.001); patients previously enrolled in the sham arm experienced a smaller increase in mean CST of 30.5 μm, from 249.4 μm (SD 52.4) to 279.9 μm (SD 50.4).

### 3.2. Clinical Course During VOYAGE

Thirty-five patients (85%, 35/41) completed the VOYAGE 1-year primary endpoint; 2 patients died (due to myocardial infarction (MI) and unknown cause), 2 were lost to follow-up, and 2 withdrew consent at week 16 and 48 due to hospitalization following a cerebrovascular accident (CVA) and patient preference, respectively. Of the 164 possible visits among all patients through the first year, 14 visits (9%) were missed.

Through 1 year of VOYAGE, 7 patients (17%, 7/41) received no 2 mg intravitreal aflibercept injections and the remaining 34 (83%, 34/41) received a mean of 3.5 injections (range 1–9 injections); among the entire population, mean injection frequency increased from 1.1 injections per year (SD 1.9) during the interim period to 3.4 injections per year (SD 2.6) during VOYAGE (*p* < 0.001) (Figure 2). Of the patients who had been in the combined aflibercept arms, 2 mg intravitreal aflibercept injection frequency increased from 1.0 injection per year (SD 1.6) during the interim to 3.4 injections per year (SD 2.7) during VOYAGE (*p* < 0.001); patients who had been in the sham arm did not experience a significant change in 2 mg intravitreal aflibercept injection frequency from the interim period (1.4 injections per year [SD 2.6]) to VOYAGE (3.3 injections per year [SD 2.2]) (*p* = 0.09).

There were no meaningful differences in the annualized mean injection frequencies between Groups 1 (3.3 injections per year [SD 2.4]) and 2 (3.7 injections per year [SD 3.5]), or among patients previously assigned to the sham, Q16W, and Q8W arms (mean of 3.3 injections per year [SD 2.2], 3.3 injections per year [SD 3.3], and 3.5 injections per year [SD 2.3], respectively) during VOYAGE. One patient (2%, 1/41) received PRP during year 1 of VOYAGE, and 1 patient (2%, 1/41) received PPV for vitreous hemorrhage.

Mean HbA1c decreased from 8.9% (SD 2.4) at VOYAGE enrollment to 8.1% (SD 1.9) at VOYAGE 1 year. Eleven patients (27%, 11/41) were on glucagon-like peptide-1 receptor agonists during VOYAGE, with no apparent meaningful differences in baseline or 1-year outcomes between these patients and the rest of the VOYAGE cohort.

#### 3.2.1. Diabetic Retinopathy Severity Scale Levels

Overall, DRSS levels appeared relatively stable through 1 year of VOYAGE; 53% (17/32) did not change DRSS level, while 28% (9/32) improved and 19% (6/32) worsened (Figure 3). The proportion of patients with DRSS level ≤ 43 decreased from 63% (25/40) to 58% (19/33), and the proportion of patients with DRSS level ≥ 60 increased from 33% (13/40) to 39% (13/33) from VOYAGE enrollment to year 1.

Of the patients who had PDR (DRSS level ≥ 60; 33%, 13/40) at VOYAGE enrollment, 42% (5/12) improved, 42% (5/12) remained stable, and 17% (2/12) worsened through 1 year. Of the patients who had NPDR (68%, 27/40) at VOYAGE enrollment, 20% (4/20) improved, 60% (12/20) remained stable, and 20% (4/20) worsened through 1 year. One of the patients (5%, 1/20) with NPDR progressed to PDR at 1 year. Four (20%, 4/20) of the patients with NPDR improved by at least 1 step by VOYAGE 1-year. Only 1 patient (3%, 1/32) developed new center-involving DME through VOYAGE 1-year.

The 6 patients (19%, 6/32) with worse DRSS levels through 1 year had mild to moderate NPDR (67%, 4/6) or inactive PDR (33%, 2/6) at VOYAGE baseline. In comparison, 26 patients (81%, 26/32) experienced DRSS level stability or improvement throughout 1 year of VOYAGE, with 23 patients (72%, 23/32) in Group 1 and 3 (9%, 3/32) in Group 2. There were no meaningful differences (*p* > 0.05) in mean HbA1c at VOYAGE initiation or 1 year, mean 2 mg intravitreal aflibercept injections received in VOYAGE at 1 year, or cumulative mean 2 mg intravitreal aflibercept injections per year since PANORAMA initiation between patients who improved or worsened in DRSS levels at VOYAGE 1-year.

#### 3.2.2. Best-Corrected Visual Acuity and Central Subfield Thickness

Mean BCVA remained stable from VOYAGE baseline (77.3 letters [SD 14.8]) to 1 year (78.9 letters [SD 17.7]) (Figure 4A). In total, 27 patients (77%, 27/35) had stable or improved BCVA through 1 year. At VOYAGE baseline, 1 patient (2%, 1/41) had a vitreous hemorrhage, resulting in 0 letters read (Figure 4B). One patient (3%, 1/35) received cataract surgery while enrolled in VOYAGE and subsequently experienced an 8-letter improvement at 1 year. At VOYAGE 1-year, 1 patient (3%, 1/35) had a traumatic cataract resulting in 0 letters read. Five patients (14%, 5/35) gained at least 10 letters from VOYAGE baseline through 1 year, and 1 patient (3%, 1/35) lost 15 letters following endophthalmitis.

At VOYAGE 1-year, mean CST decreased from 293.9 μm (SD 67.9) to 269.5 μm (SD 34.5) (*p* = 0.06) (Figure 4C). Twenty-one patients (64%, 21/33) had improved CST through 1 year. There was no significant difference in CST decrease among patients who had PDR (46.2 μm [SD 105.3]) versus NPDR at VOYAGE baseline (10.6 μm [SD 40.0]), or between Group 1 (22.3 μm [SD 71.3]) and Group 2 (32.3 μm [SD 84.0]) patients. Patients previously assigned to sham, Q16W, and Q8W during PANORAMA had mean decreases in CST of 13.8 μm (SD 36.1), 13.7 μm (SD 62.4), and 36.8 μm (SD 93.6), respectively, which also did not significantly differ from one another.

#### 3.2.3. Adverse Events

Through 1 year of VOYAGE, treatment-emergent AEs occurred in 28 patients (68%, 28/41), none of which were determined to be related to aflibercept. There was 1 case of traumatic cataract related to the injection procedure at week 48 out of 119 total injections (incidence of 0.8%), which included 72 injections in phakic eyes (incidence of 1% of phakic injections), during the prospective component of VOYAGE. Inflammatory events were reported in 2 patients (5%, 2/41), with 1 case of concurrent anterior chamber cells and vitreous haze, which were reportedly related to keratoconjunctivitis sicca and active PDR, respectively, and did not require treatment, and 1 case of endophthalmitis (incidence of 1/119 injections, or 0.8%) reported as related to the injection procedure. Reported serious AEs included 1 vascular death from a fatal MI (2%, 1/41), 1 death of unknown cause (2%, 1/41), and 1 nonfatal CVA (2%, 1/41).

## 4. Discussion

Results from the prospective VOYAGE study, which enrolled a subset of patients who completed the PANORAMA phase 3 trial, indicate that during an approximate 3-year period of routine clinical care, DRSS level, CST, and BCVA all appeared to numerically worsen, particularly among patients who had been randomized to receive 2 mg intravitreal aflibercept injection during PANORAMA. Concurrently, there was a decrease in intravitreal anti-VEGF injections administered during the interim period. Then, following initiation of aflibercept therapy for pre-defined DR activity thresholds during VOYAGE, overall mean BCVA and CST increased by 1.8 letters and decreased by 24.4 µm, respectively. Relevant to this reversal, patients received intravitreal anti-VEGF injections more than 4 times as frequently during PANORAMA (4.8 injections per year) and almost 3 times as frequently during VOYAGE (3.4 injections per year) compared to the interim period (1.1 injections per year).

From PANORAMA baseline to the VOYAGE 1-year timepoint, DRSS levels worsened for many patients, with 39% (13/33) of patients developing PDR. This DRSS level worsening occurred predominantly during the interim period, when less frequent anti-VEGF treatment was utilized, and DRSS levels predominantly stabilized with more frequent treatment during VOYAGE. More frequent anti-VEGF dosing may have been needed to achieve maximal DRSS level improvement. Furthermore, the ability to improve DRSS levels may be at least partially dependent on initial disease severity; specifically, in some datasets, DRSS level improvements have been less robust among patients with PDR compared to those with NPDR [12,13]. For example, in CLARITY, 78% of patients treated with a mean of 4.4 aflibercept injections through 1 year did not improve to NPDR [12]; VOYAGE seemed to produce similar results, with 11 of the patients (92%, 11/12) with PDR not returning to NPDR following 1 year of aflibercept treatment. Additionally, there were no meaningful differences in mean HbA1c at VOYAGE baseline or 1 year between patients who experienced improved or worsened DRSS levels at VOYAGE 1-year. Larger studies with longer follow-up time are needed to better identify patient-level factors that may influence who benefits most from sustained aflibercept therapy.

Considering BCVA and CST longitudinally, although both appeared relatively stable between PANORAMA baseline and the VOYAGE 1-year timepoint, there were periods of substantial fluctuation throughout the 5.7 years in between. These changes appear to have been driven by the meaningfully different approaches to clinical care utilized during these 3 time periods. Specifically, during the interim period when patients received routine care outside of a rigid prospective protocol, VA and CST temporarily worsened by over 6 ETDRS letters and 58 µm, respectively. Of note, fluctuations in specific DME biomarkers have been reported to be associated with worse visual outcomes. For example, greater CST fluctuations have been associated with worse VA outcomes at 1 and 2 years among DME patients in retrospective analyses [14,15]. While the visit-to-visit granularity of CST fluctuations is beyond the scope of the current work, overall data suggests that in order to maximize clinical outcomes, it may be worthwhile to consider a personalized approach with more consistent intervention in order to minimize disease fluctuations.

During the interim period, when less structured follow-ups and less consistent anti-VEGF dosing were utilized, clinical characteristics appeared to worsen. According to the 2024 American Society of Retina Specialists Preferences and Trends Survey, inconsistent or insufficient office visits were found to be the factor that has the greatest negative impact on all patient outcomes in the United States [16]. However, providing consistent anti-VEGF therapy to patients in a real-world setting is challenging due to a multitude of factors [17]. Additionally, intravitreal injections carry risk as evidenced by the cases of infectious endophthalmitis and traumatic cataract observed in the current series. In order to address these meaningful hurdles to optimal outcomes and care delivery, multiple therapeutic approaches currently under investigation seek to provide consistent, longitudinal ocular-specific treatment for DR and other chronic, exudative retinal diseases. These include anti-VEGF port delivery systems [18], extended durability agents such as tyrosine kinase inhibitors [19,20], and anti-VEGF gene therapies such as RGX-314 and ADVM-022, both of which have demonstrated durable intraocular anti-VEGF protein production and exudative disease control following subretinal and intravitreal delivery, respectively [21,22,23]. For any potential more durable therapy, the risks and benefits will need to be considered in the clinical context of each patient.

Limitations of the current work include that of the 320 patients across 5 countries who completed PANORAMA, only 41 patients (13% of PANORAMA completers) enrolled in VOYAGE, and all patients were from United States sites, which may limit the generalizability of the results. The underlying reasons for this are likely multifactorial, including study-site operational limitations, patients lost to follow-up, and the additional significant time commitment after having already participated in PANORAMA. While this low VOYAGE enrollment number raises concerns about selection bias and whether this cohort accurately reflects the entire PANORAMA patient population, no meaningful differences were observed in PANORAMA baseline characteristics between the subset that enrolled in VOYAGE and the full PANORAMA cohort. Additionally, although there were no apparent outcome differences between Group 1 and Group 2 patients, the cohort of patients in Group 2 was small (*n* = 6), which limited the overall ability to meaningfully compare these groups.

Additionally, a key challenge when assessing aflibercept efficacy and durability are the specific criteria used to determine when and how often to treat. VOYAGE treatment criteria were designed to allow physicians to use clinical judgment as to the necessity of retreatment in cases of DME. While this better reflects a real-world approach, it also generates greater variability in treatment decisions and therefore may have influenced variability in outcomes. Future studies using standardized treatment criteria may reduce this variability and improve consistency among outcomes. Another interesting result from this real-world approach was the observation that, on an individual-patient basis, clinician-determined DRSS levels and reading center-determined DRSS levels sometimes disagreed; whereas investigators’ scores were distributed more evenly across all NPDR severity levels, the reading center leaned heavily toward mild NPDR scores. Across all patients enrolled, comparing investigator to reading center scores did not change the results, particularly regarding the number of PDR patients. This discrepancy may be due to various factors, such as access to information or time constraints in the clinic setting.

Another key limitation is that because the treatment regimens during the interim period varied considerably, it is challenging to compare how much of the observed anatomic and functional decline was attributable to undertreatment versus disease progression. Additionally, all analyses utilized observed data, so meaningful outliers may have impacted mean values. For example, true mean BCVA improvement may have been underestimated due to the inclusion of 1 case (3%, 1/35) of 0 letters read at 1 year due to traumatic cataract, resulting in an apparent 75 letter loss from VOYAGE baseline through 1 year. Outliers were described when results were meaningfully impacted.

In summary, the observed changes in ocular characteristics across the mean 5.7 years from PANORAMA enrollment through the VOYAGE 1-year endpoint appeared to correlate with anti-VEGF dosing frequency. Patients who received less frequent 2 mg intravitreal aflibercept injections after PANORAMA exit experienced a trend toward worsening of DRSS level, BCVA, and CST at VOYAGE enrollment, trends which appeared to stabilize or improve after re-initiating regular 2 mg intravitreal aflibercept injections during VOYAGE. More data on long-term outcomes are needed from larger patient populations with NPDR to assess the sustainability of these treatment effects and to adequately inform clinical management of this common, chronic condition. Finally, new, safe therapeutics which meaningfully reduce treatment burden may be valuable for personalized, long-term DR management.

## Figures and Tables

**Table 1:** Demographic and ocular characteristics of patients in VOYAGE and PANORAMA.

	Prospective VOYAGE Enrollment *		PANORAMA Enrollment ^†^			Enrollment Baseline Totals	
	Group 1 (*n* = 35)	Group 2 ^§^ (*n* = 6)	Sham (*n* = 11)	Q16W ^¥^ (*n* = 12)	Q8W (*n* = 18)	VOYAGE ^‡^ (*n* = 41)	PANORAMA (*n* = 402)
**Age, mean (SD), yrs**	59.4 (12.0)	59.0 (6.3)	63.4 (8.9)	58.1 (14.7)	57.6 (10.0)	59.3 (11.3)	55.7 (10.5)
**Male, *n* (%)**	19 (54)	2 (33)	5 (46)	6 (50)	10 (56)	21 (51)	225 (56)
**Race, *n* (%)**							
** White**	30 (86)	6 (100)	10 (91)	12 (100)	14 (78)	36 (88)	310 (77)
** Black**	4 (11)	0	1 (9)	0	3 (17)	4 (10)	41 (10)
** Asian**	1 (3)	0	0	0	1 (6)	1 (2)	23 (6)
** Other**	0	0	0	0	0	0	28 (7)
**Hispanic or Latino, *n* (%)**	19 (54)	1 (17)	7 (64)	6 (50)	7 (39)	20 (49)	Not reported
**HbA1c, mean (SD), %**	8.6 (2.2)	10.2 (3.0)	8.9 (2.7)	8.4 (2.3)	9.2 (2.3)	8.9 (2.4)	8.5 (1.6)
**ETDRS letters, mean (SD)**	79.3 (8.7)	65.5 (32.2)	82.2 (6.8)	73.8 (23.8)	76.7 (9.9)	77.3 (14.8)	82.4 (6.0)
**Snellen equivalent**	20/25	20/50	20/25	20/32	20/32	20/32	20/25
**CST, mean (SD), µm**	291.9 (67.7)	308.0 (75.4)	279.9 (50.4)	294.9 (42.8)	301.9 (88.4)	293.9 (67.9)	247.4 (34.8)
**DRSS level, *n* (%)**							
** ≤43**	25 (71)	0	6 (55)	6 (50)	13 (72)	25 (61)	0
** 47**	1 (3)	0	0	1 (8)	0	1 (2)	302 (75)
** 53**	1 (3)	0	0	1 (8)	0	1 (2)	100 (25)
** ≥60**	8 (23)	5 (100)	5 (45)	3 (25)	5 (28)	13 (32)	0

CST = central subfield thickness; DRSS = diabetic retinopathy severity scale; ETDRS = Early Treatment Diabetic Retinopathy Study; HbA1c = glycosylated hemoglobin; SD = standard deviation. * Patients assigned to Group 1 (no history of panretinal photocoagulation) and Group 2 (history of panretinal photocoagulation) during VOYAGE. ^†^ Patients initially randomized to the sham, every-16-week (Q16W), and every-8-week (Q8W) arms during PANORAMA. ^‡^ CST *n* = 40 due to 1 case of vitreous hemorrhage at VOYAGE baseline; DRSS *n* = 40 due to 1 case of DRSS level not measured at VOYAGE baseline. ^§^ DRSS *n* = 5 due to 1 case of DRSS level not measured at VOYAGE baseline. ^¥^ DRSS *n* = 11 due to 1 case of DRSS level not measured at VOYAGE baseline.

## Data Availability

The data collected in the course of this study is not publicly available due to it containing information that could compromise the privacy of research patients. Further inquiries can be directed to the corresponding author.

## Supplementary Materials

The following supporting information can be downloaded at: https://www.mdpi.com/article/10.3390/jpm15110555/s1, Table S1. Participating sites in PANORAMA and VOYAGE.

## Abbreviations

The following abbreviations are used in this manuscript:

AE	Adverse event
ANOVA	Analysis of variance
BCVA	Best-corrected visual acuity
CST	Central subfield thickness
CVA	Cerebrovascular accident
DME	Diabetic macular edema
DR	Diabetic retinopathy
DRSS	Diabetic retinopathy severity scale
ETDRS	Early treatment diabetic retinopathy study
HbA1c	Glycosylated hemoglobin
MI	Myocardial infarction
NPDR	Non-proliferative diabetic retinopathy
OCT	Optical coherence tomography
PDR	Proliferative diabetic retinopathy
PPV	Pars plana vitrectomy
PRN	Pro re nata (as needed)
PRP	Panretinal photocoagulation
Q16W	Every-16-week aflibercept following 3 initial monthly doses and one 8-week interval through 2 years
Q8W	Every-8-week aflibercept following 5 initial monthly doses with pro re nata dosing in year 2 if diabetic retinopathy severity scale level was worse than 35
SD	Standard deviation
VA	Visual acuity
VEGF	Vascular endothelial growth factor
