# Supplementary material for: Intravitreal Aflibercept for the Treatment of Diabetic Retinopathy Among Patients Who Completed PANORAMA: 1-Year Outcomes from the VOYAGE Extension Study
Source: J Pers Med. 2025 Nov 14;15(11):555. doi: 10.3390/jpm15110555 (PMC12653527; doi:10.3390/jpm15110555)
Supplement: Supplementary file 1 [file jpm-15-00555-s001.zip › jpm-3894084-supplementary.pdf]

Table S1. Participating sites in PANORAMA and VOYAGE.

Site	Principal Investigator	PANORAMA Completions	VOYAGE Enrollments
Retina-Vitreous Associates Medical Group	David S. Liao, MD	4	1
Retina Consultants of Texas	James C. Major, Jr., MD	10	3
Palmetto Retina Center	W. Lloyd Clark, MD	5	2
Palmetto Retina Center	John F. Payne, MD	3	1
Strategic Clinical Research Group	Seong Y. Lee, MD	9	3
Valley Retina Institute	Victor H. Gonzalez, MD	7	2
Center for Retina and Macular Disease	Adam S. Berger, MD	15	2
Dean McGee Eye Institute	Ronald M. Kingsley, MD	4	2
John-Kenyon American Eye Institute	Howard S. Lazarus, MD	4	1
Charles Retina Institute	Stephen M. Huddleston, MD	8	1
Central Florida Retina	Eric G. Feinstein, MD	3	1
Cumberland Valley Retina Consultants	Allen Y. Hu, MD	23	6
Marietta Eye Clinic	Annal D. Meleth, MD	3	1
Emanuelli Research and Development Center	Andres Emanuelli, MD	27	15
**Total Patients**		125	41
